# MET Expression in Primary and Metastatic Clear Cell Renal Cell Carcinoma: Implications of Correlative Biomarker Assessment to MET Pathway Inhibitors

**DOI:** 10.1155/2015/192406

**Published:** 2015-09-13

**Authors:** Brian Shuch, Ryan Falbo, Fabio Parisi, Adebowale Adeniran, Yuval Kluger, Harriet M. Kluger, Lucia B. Jilaveanu

**Affiliations:** ^1^Department of Urology, Yale School of Medicine, New Haven, CT 06520-8058, USA; ^2^Yale Cancer Center, Yale University School of Medicine, New Haven, CT 06520-8028, USA; ^3^Department of Pathology, Yale University School of Medicine, New Haven, CT 06520-8023, USA

## Abstract

*Aims*. Inhibitors of the MET pathway hold promise in the treatment for metastatic kidney cancer. Assessment of predictive biomarkers may be necessary for appropriate patient selection. Understanding MET expression in metastases and the correlation to the primary site is important, as distant tissue is not always available. *Methods and Results*. MET immunofluorescence was performed using automated quantitative analysis and a tissue microarray containing matched nephrectomy and distant metastatic sites from 34 patients with clear cell renal cell carcinoma. Correlations between MET expressions in matched primary and metastatic sites and the extent of heterogeneity were calculated. The mean expression of MET was not significantly different between primary tumors when compared to metastases (*P* = 0.1). MET expression weakly correlated between primary and matched metastatic sites (*R* = 0.5) and a number of cases exhibited very high levels of discordance between these tumors. Heterogeneity within nephrectomy specimens compared to the paired metastatic tissues was not significantly different (*P* = 0.39). *Conclusions*. We found that MET expression is not significantly different in primary tumors than metastatic sites and only weakly correlates between matched sites. Moderate concordance of MET expression and significant expression heterogeneity may be a barrier to the development of predictive biomarkers using MET targeting agents.

## 1. Introduction

Small molecule tyrosine kinase inhibitors of the vascular endothelial growth factor receptors have been widely used as a standard of care for metastatic renal cell carcinoma (RCC) since FDA approval of the multiprotein kinase inhibitors, sunitinib, and sorafenib in early 2006 [1–3]. Since then, multiple new agents targeting this pathway have been developed and are in clinical use. While these agents may provide clinical benefit to patients, complete responses are exceedingly rare and are not durable and thus more effective agents are immediately needed [4, 5].

The MET pathway is constitutively phosphorylated/activated with loss of the VHL protein [6], a feature present in over 80% of cases of clear cell RCC (ccRCC) [7, 8].* In vitro* studies have demonstrated that with VHL loss, activation of the MET pathway drives a more invasive phenotype [9]. Moreover, preclinical models of resistance to VEGFR-directed therapy are believed mediated by the Hepatocyte Growth Factor Receptor (MET) pathway [10]. MET might therefore be an important therapeutic target in ccRCC.

Limited data exists on the relationship of MET expression and prognosis in ccRCC. Clear cell renal tumors can have wide variability in MET expression. However, those with increased protein and mRNA levels have more aggressive pathologic characteristics and worse prognosis [11–13].* In vitro* targeted inhibition of MET in ccRCC cell lines, in which its expression is upregulated, decreases proliferation and colony formation [11], providing rationale to block this pathway either alone or in conjunction with the VEGFR pathway.

Multiple therapeutic strategies have been developed to block the MET pathway including several small molecule inhibitors and antibodies [14]. MET pathway inhibitors have been studied in kidney cancer. AMG102, a monoclonal antibody to the ligand of MET, Hepatocyte Growth Factor (HGF) was studied in a phase II trial but had limited efficacy with progression-free survival (PFS) of less than 4 months [15]. A tyrosine kinase inhibitor to VEGFR2 and MET, cabozantinib (XL184), was studied in a small phase I trial for RCC and later gained FDA approval for medullary thyroid cancer. Despite enrolling a heavily pretreated RCC population, there was significant antitumor activity with a 28% response rate and a 12.9-month PFS [16]. Further phase III studies with this FDA approved agent are currently ongoing in the first and second line metastatic setting.

In the era of targeted therapy, response may be dictated on whether the actual therapeutic target is present in the cancer cell. Therefore, the presence of an upregulated, overexpressed, or mutated pathway may serve as a useful predictive biomarker. Adaptive biomarker trials have become more common in recent years as clinicians have tried to match patients with an appropriate therapy. Previous studies have shown that MET expression in clear cell RCC can be variable [11], something that may influence therapeutic response. These studies, however, focused on expression in primary RCC specimens, while expression in corresponding metastatic tumors has not yet been characterized. In this study, we investigate the expression and correlation of MET in matched metastatic and primary clear cell renal tumors in order to aid future efforts to predict clinical response based on tissue expression.

## 2. Materials and Methods

### 2.1. Tissue Microarray (TMA) Construction

With Institutional Review Board approval (HIC #9505008219/2014), we reviewed charts of patients treated at Yale University between 1972 and 2011. A TMA was created from a cohort of thirty-four patients and all patients had matched nephrectomy and metastasectomy specimens. Patient and tumor characteristics and other clinical information have been described previously [17, 18]. Briefly, all patients had clear cell histology; however three (9%) had regions of sarcomatoid transformation. Four punches from each specimen and cell pellet controls were placed on separate blocks as previously detailed [17, 18].

### 2.2. Immunofluorescence and Automated Quantitative Analysis (AQUA)

TMA slides were deparaffinized and processed for antigen-retrieval. Endogenous peroxidase activity was blocked before overnight incubation with MET4, a mouse anti-c-Met antibody (1 : 7500 dilution; kindly provided by Dr. George Vande Woude, Grand Rapids, MI). This antibody was validated and utilized in a previous study [11]. Anti-mouse secondary antibody (Envision, Dako North America, Inc., Carpinteria, CA) was used along with cyanine-5-tyramide (Cy5; Perkin Elmer, Inc., Waltham, MA) for signal amplification. A tumor mask was created by incubation with rabbit anti-cytokeratin (1 : 100 dilution; Cat. Number M5315, Dako) for 2 hours at room temperature. A goat anti-rabbit HRP-decorated polymer backbone (Envision, Dako) was used as a secondary reagent. Incubation with cyanine 2-tyramide (Cy2, Perkin Elmer, Inc., Waltham, MA) was used to visualize tumor mask. A nuclear mask was created by incubating with 4, 6-diamidine-2-phenylindole (DAPI) (Invitrogen, Carlsbad, CA, dilution 1 : 500). Coverslips were mounted with ProLong Gold antifade medium (Invitrogen/Life Technologies TM, Grand Island, NY).

### 2.3. Automated Image Acquisition and Analysis

High-resolution (1024 × 1024 pixels) images were obtained of each histospot as previously described [19]. In brief, monochromatic grayscale images were acquired with a 10x objective of an Olympus AX-51 epifluorescence microscope (Olympus) operating via an automated microscope stage. Digital image acquisition is driven by a custom program and macrobased interfaces with IPLabs software (Scanalytics, Inc.). For the tumor mask, we used the Cy2 signal while DAPI was used to identify the nuclei. The tumor mask is a binary image created from the cytokeratin image (Cy2 signal) of each histospot. DAPI images were used to create the nuclear compartment within each histospot. The membrane compartment within the tumor mask was defined by the perimembranous coalescence of cytokeratin signal with specific exclusion of the nuclear compartment. MET signal was visualized by Cy5, compartmentalized, and expressed as the average signal intensity within the assayed component (AQUA score), with scores on a scale of 0–255.

### 2.4. Data Analysis

JMP 5.0 software was used for analysis (SAS Institute, Cary, NC). Associations between continuous AQUA scores and clinical/pathological parameters were assessed by analysis of variance. Correlations between the AQUA scores of matched primary and metastatic histospots were calculated paired sample* t* and chi-squared testing. For heterogeneity assessment between cases, a composite median absolute deviation (MAD) score was generated for each tumor site by patient and compared using a Wilcoxon paired, two-sided rank test, as previously described [20].

## 3. Results

To compare MET expression in primary and metastatic RCC tumors and evaluate expression heterogeneity, MET expression was quantitatively assessed on a custom TMA previously described [17, 18]. Briefly, the array was constructed with paired primary tumors and distant sites of metastasis and contained a total of eight cores for each patient (four primary and four metastatic) distributed across two blocks.

**Figure 1:**
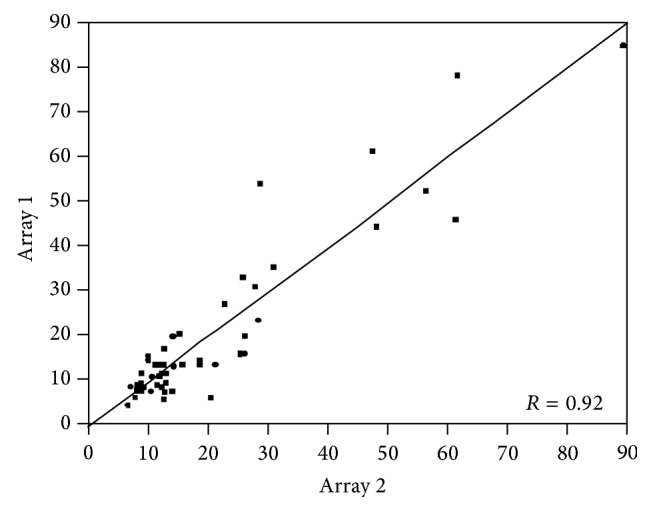
Correlation of MET expression between the two array blocks (*R* = 0.92). Pearson correlation test was used to compare scores from the two tissue microarrays stained for MET (*R* = 0.92).

As was previously reported, MET staining was predominantly cytoplasmic [11]. To assess the interarray variability of MET expression, the correlation between AQUA scores from corresponding cores (from the same tumor block) from each array was analyzed by linear regression. We found that the scores on the two arrays were highly correlated as demonstrated in Figure 1 (*R* = 0.92).

**Figure 2:**
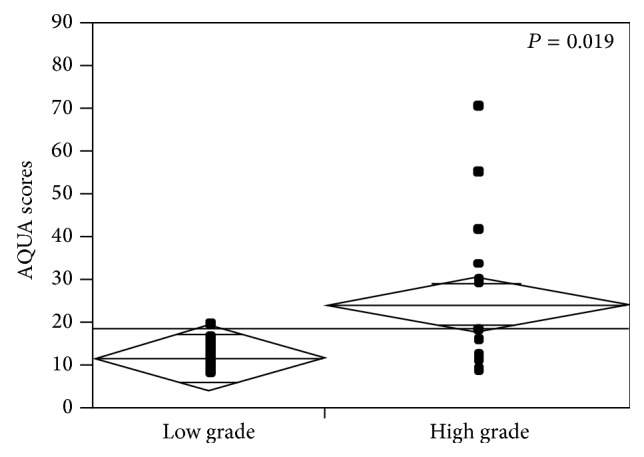
MET expression in low and high Fuhrman grade tumors (1/2 versus 3/4).* t*-test was used to compare the means of MET expression (AQUA scores) in “low” (1 and 2) versus “high” (3 and 4) Fuhrman grade tumors (*P* = 0.019).

Using a larger RCC TMA we previously demonstrated an association between MET expression and Fuhrman grade [11]. By using the paired sample* t-*test, we confirmed this association in this small cohort of predominantly clear cell RCC cases. As shown in Figure 2, the mean expression of MET was over two times greater in high grade tumors (grades 1/2 versus 3/4; mean AQUA scores of 11.825 and 24.258, resp.;* P* = 0.019). For the mixed histology cases, no significant differences were seen between MET expression in tissue with sarcomatoid features and tissue without.

**Figure 3:**
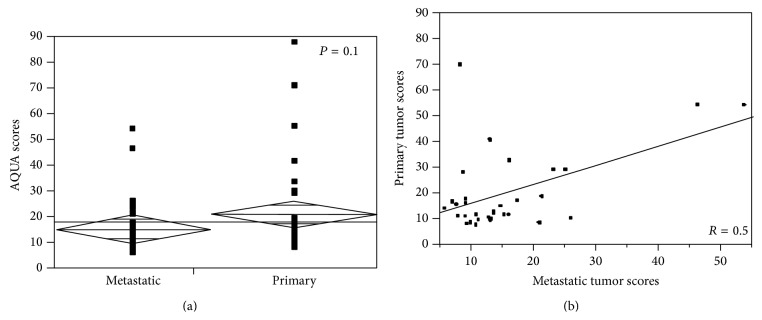
(a) MET expression levels in metastatic versus primary tumors.* t*-test was used to compare the means of MET expression (AQUA scores) in matched primary and metastatic specimens from the same patients (*P* = 0.1). (b) Correlation of MET expression between primary tumor and matched metastases. Pearson correlation test was used to measure the degree of correlation between MET expressions (AQUA scores) in matched primary versus metastatic specimens (*R* = 0.5).

AQUA scores ranged from 7.815 to 87.370 for primary RCC tissue and from 5.705 to 53.843 for metastatic tissue. To compare MET expression between nephrectomy specimens and metastatic RCC tissues, we used* t-*test. The mean expression of MET in primary tumors (mean AQUA scores for all four cores) was not significantly different when compared to their metastatic counterparts (mean AQUA score of 21.23 versus 15.35, resp.,* P* = 0.1, Figure 3(a)).

**Figure 4:**
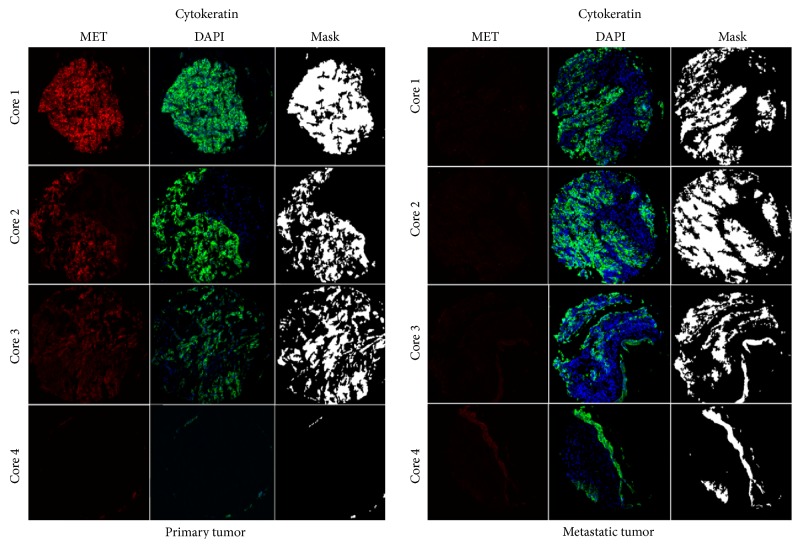
Quantitative immunofluorescent staining of MET in matched primary and metastatic cores. We utilized MET4 antibody to determine expression of MET in a cohort of matched primary and metastatic RCC cases. Cytokeratin (Cy2 signal) was utilized to create a tumor mask. DAPI was used to identify the nuclei. MET signal was visualized by Cy5 and intensities of MET expression were measured within the cytokeratin mask, within the histospot in a quantitative fashion.

Seeing that archival primary RCC tissue is often more readily available to determine patient eligibility for targeted therapy, we studied the correlation of MET expression between primary sites and paired metastatic tissues using the Pearson correlation test. As shown in Figure 3(b), MET expression correlated between the primary and metastatic sites, although the correlation coefficient was modest (*R* = 0.5). While some cases had a low level of discordance, a number of cases exhibited very high levels of discordance. One such example is shown in Figure 4; three out of three assessable primary tumor cores showed either high or moderate staining, while none of the four metastatic cores showed any detectable levels of MET expression. Analysis of scores dichotomized by the median into “high” and “low” showed that in only 58% of cases (18 of 31 assessable cases) scores were concordant, while 42% of the cases (13 of 31) were discordant (Table 1). By chi-square analysis, there was no significant difference between the distribution of high and low MET expression and the two tumor types (primary versus metastatic) (*χ* ^2^ *P* = 0.375).

**Figure 5:**
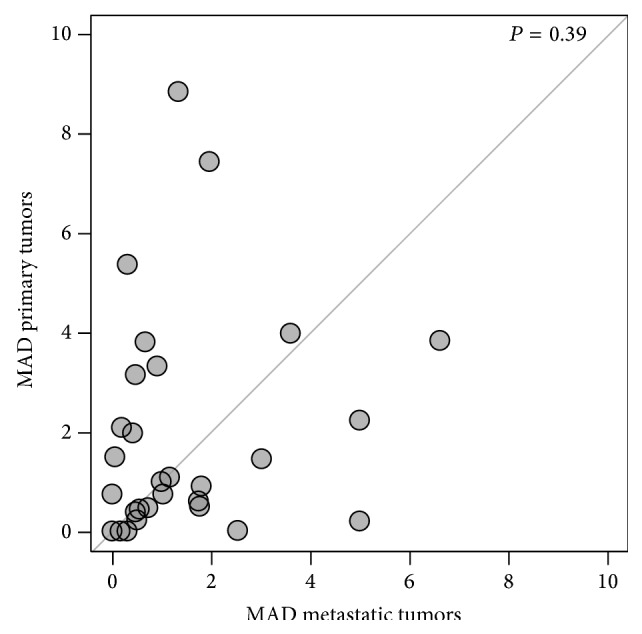
Assessment of MET staining heterogeneity by MAD score for primary and metastatic lesions. We employed the composite median absolute deviation (MAD) to estimate the heterogeneity within primary and metastatic specimens in our cohort. Cases (dots) with larger primary tumor heterogeneity are above the diagonal, while those with a greater heterogeneity in the corresponding metastatic tumors are below the diagonal.

To estimate the degree of heterogeneity of MET expression in primary and metastatic sites, we used the scores from the four cores for each tumor and determined the median absolute deviation (MAD). As seen in Figure 5, a wide range of MAD scores was obtained for each case, indicating wide variability in the degree of heterogeneity. The difference between heterogeneity within nephrectomy specimens compared to the paired metastatic tissues was not statically significant by virtue of a Wilcoxon paired, two-sided rank test (*P* = 0.39).

## 4. Discussion

With multiple agents now available in the current era of kidney cancer treatment, selection and sequencing is becoming a challenge for clinicians. In order to investigate predictive biomarkers of clinical response, many trials involving targeted agents have incorporated access to resected tissue into the trial eligibility. Two ongoing trials (METEOR, NCT01865747 and CABOSUN, NCT01835158) evaluate XL184 in patients with clear cell RCC, both of which require tissue submission from either the primary tumor or a site of metastasis for retrospective correlative biomarker analysis. As many patients have had a prior nephrectomy, this tissue is more readily available for study than a distant site, which is generally assessed with a small core biopsy. With increasing recognition of tumor clonality in kidney cancer, it is necessary to understand if the correlative biomarker analyses should be performed on tissue that represents the metastatic disease. Our data indicate that expression of MET in primary sites does not necessarily correlate with expression at distant sites. While studies have investigated MET expression in the primary clear cell RCC [11, 21], no study to date has evaluated MET expression in a cohort of distant sites or compared matched primary tumor and metastases. We investigated this question using TMAs and a method of quantitated immunohistochemistry (AQUA).

While there are multiple commercially available MET antibodies, many have been reported to have insufficient sensitivity and specificity for biomarker analysis [22]. The antibody we utilized for this study was created by Cao et al. [23]. Knudsen and colleagues interrogated this antibody's performance and found it has excellent technical reproducibility and improved sensitivity when compared to commercially available products [24]. Our analysis of the MET4 antibody confirms the extremely high correlation between arrays (*R* = 0.92).

Limited data exists to demonstrate a correlation between increased MET expression and aggressive pathologic characteristics or/and worse prognosis in RCC. Similar to previous studies, we found that high grade tumors have a greater degree of MET expression (*P* = 0.019). This result supports work from our group and Choi et al. demonstrating tumors with increased MET expression are associated with higher grade and stage disease [11, 25]. While this association has been shown in multiple tumor types, in kidney cancer, there has been some conflicting data including that from Miyata and colleagues [21]. In this analysis, total MET expression was not associated with clinicopathologic characteristics; however pMET was associated with advance stage, higher grade, and the presence of metastatic disease [21]. Differences in these findings could be due to the fact that different antibodies were used between studies. Additionally we did not examine a pMET antibody in our cohort due to the difficulty with signal preservation in formalin-fixed paraffin embedded tissue.

When treating systemic disease with molecular targeted therapy, one would hope the target is highly expressed outside the primary tumor and that the degree of expression would predict response. Our data is the first to evaluate MET expression in sites of distant RCC. We demonstrated that MET expression was not significantly different in metastases when compared to primary sites. For trials involving MET inhibitors, correlative biomarkers are planned on the available tissue, which has generally been submitted from the nephrectomy specimen. While there has been renewed interest in neoadjuvant approaches in locally advanced tumors [26], the majority of systemic therapy in RCC is used to treat distant disease. Therefore correlative biomarker analyses should focus on tissue obtained from distant sites unless the local tumor expression was perfectly correlated with distant disease. In our analysis of the matched sites an important observation was seen; that is, over 40% of specimens had discordant expression when dichotomized to high versus low expression. Also there was only a low level of correlation between MET expressions between the primary and distant tissue (*R* = 0.5). Based on these findings, caution should be used when interpreting correlation of systemic response to MET therapy if MET expression was generally obtained from tissue from the primary tumor.

Tumor heterogeneity is a concern for any biomarker that may affect treatment, particularly, if the biomarker is to be assessed using tissue from core needle biopsies, which are similar in diameter to our TMA histospots. While the primary tumors often had more MET expression heterogeneity, overall there was no significant difference between sites. We found that MET expression heterogeneity was occasionally high in both the primary tumor and metastatic sites. This raises some concern that sampling expression on limited amount of tissue such as a small core biopsy of either the primary or a distant site may not represent the biology of the majority of the tumor.

Our findings represent the first effort to characterize MET expression patterns in primary and metastatic clear cell RCC. The strengths of this study involve our unique TMA design of matched sites of disease and a novel method of quantitative immunohistochemistry (AQUA). Limitations include the relatively small number of samples, the use of various sites of metastatic disease, and our inability to study pMET in this cohort.

## 5. Conclusions

MET is a therapeutic target in clear cell RCC and selective inhibitors are currently in clinical trials. Studies of biomarkers predictive of response are planned in many of these trials with the goal of improving the therapeutic window of these inhibitors. Here we demonstrate a weak positive correlation between MET expressions in matched primary and metastatic sites. Moderate concordance of high and low levels of MET expression and significant expression of heterogeneity may be a barrier to the adoption of tissue biomarkers assessing MET expression. Prospective validation of our findings is warranted, as agents targeting the MET pathway appear promising in this disease.

## Figures and Tables

**Table 1:** MET expression in primary and metastatic renal cell carcinoma (RCC).

	Primary RCC		
	High MET # (%)	Low MET # (%)	Total cases
Metastatic RCC			
High MET # (%)	10 (58.8%)	6 (42.9%)	16
Low MET # (%)	7 (41.2%)	8 (57.1%)	15
Total cases	17	14	31
